# Adaptation of the Nurse Practitioner Integration Model for older adult primary care models

**DOI:** 10.1016/j.gerinurse.2025.103453

**Published:** 2025-07-09

**Authors:** Joshua Porat-Dahlerbruch, Ivy Chen, Katherine Cahir, Nancy P. Blumenthal

**Affiliations:** a Department of Acute and Tertiary Care, School of Nursing, University of Pittsburgh, 336 Victoria Building, 3500 Victoria Street, Pittsburgh 15090, PA, USA; b School of Nursing, University of Pennsylvania, 418 Curie Blvd., Philadelphia 19104, PA, USA

**Keywords:** Advanced practice nursing, Aged, Consensus, Geriatrics, Implementation, Models, Nurse practitioners, Nursing theory

## Abstract

To reap positive outcomes associated with nurse practitioner care, nurse practitioners must be integrated efficaciously into the care team. However, the process has been described inconsistently, creating a lack of clarity for research and applied use in practice. The Nurse Practitioner Integration Model was developed to provide a consistent conceptualization and description of the process of implementing and incorporating nurse practitioners into care teams in any setting. The model needs to be honed for use in older adult primary care settings. This study aimed to adapt the Nurse Practitioner Integration Model for older adult primary care settings. A qualitative, nominal group consensus technique was used. Experts discussed, debated, and reached consensus on an adapted model. The model consists of a revised definition of nurse practitioner integration, an illustrative model, precursors, outcomes, and attributes. Integration mediates introducing nurse practitioners and care outcomes. Empirical research is needed to validate the model.

## Introduction

The older adult population is expected to increase from today’s 56 million to 78 million by 2030.^1,2^ Older adults have the highest prevalence of multiple chronic conditions.^3^ Poorly managed chronic conditions and the consequential costly hospitalizations are linked to inadequate primary care access.^4^ To improve patient outcomes and reduce system costs, it is prudent to ensure adequate access to primary care for older adults.

Nevertheless, increasing demand for primary care services, driven by the aging population, is outpacing supply. One in four Americans lacks a primary care provider.^5^ A national shortage of 35,000 primary care providers is projected in 2035.^6^ Government incentive programs tend to focus on growing primary care physician supply, though historically have been unsuccessful at achieving sustainable growth over time.^7^ Focusing on non-physician providers, such as nurse practitioners (NPs),^1^ may be a more sustainable solution.^8,9^

There are about 350,000 NPs, and 70 % of them deliver primary care services.^10^ A recent systematic review of outcomes among patients with multiple chronic conditions, most of whom are older adults, found that NP-inclusive primary care models, compared to models without NP involvement, resulted in improved care access, similar costs, equivalent or better quality, similar or lower rates of emergency department use, and lower or similar hospitalization rates.^4^ To reap these positive benefits of NP care for older adults, NPs must be integrated efficaciously into primary care models.^11–13^

The integration of NPs can be defined as, “a multi-level process of incorporating NPs into the health system to an extent at which they can function to their full scope of practice and education and contribute to patient, health system, and population needs.”^14^ Policy decision-makers (i.e., organizational managers and policymakers) lack knowledge of the best practices for integrating NPs into care models.^8,11^ Until recently, there were no frameworks nor models to guide research on NP integration. Understanding systematic approaches, such as through evidence-based models, for maximizing the contribution of NPs to older adult primary care is essential considering the shortage of providers specializing in older adult primary care and the increasing demand for older adults to receive care management in community-based primary care practices.^15^

The NP Integration Model was published in 2022 to fill these gaps in policy and research.^14^ The model was based on a literature review and concept analysis. It describes antecedents, consequences, attributes, an illustrative model, definition, and factors affecting NP integration. The model spans three health system levels (i.e., *macro* (national/regional), *meso* (organizational), and *micro* (care team) levels).^11^

The model, however, is conceptual. While a few studies have cited the model, it lacks further investigation to validate its contents.^12,16,17^ Moreover, the model was based on integrating NPs from all specialties. There may be variation in integration patterns across different care settings, such as older adult primary care settings. Accordingly, further model refinement is needed to move this conceptual and theoretical work toward operationalization in older adult primary care settings.^18–21^ A common approach to bridge conceptual development to empirical testing is expert consensus studies. This study aimed to adapt the NP Integration Model for older adult primary care settings through expert consensus.

## Materials and methods

### Study design

**Fig. 1.**
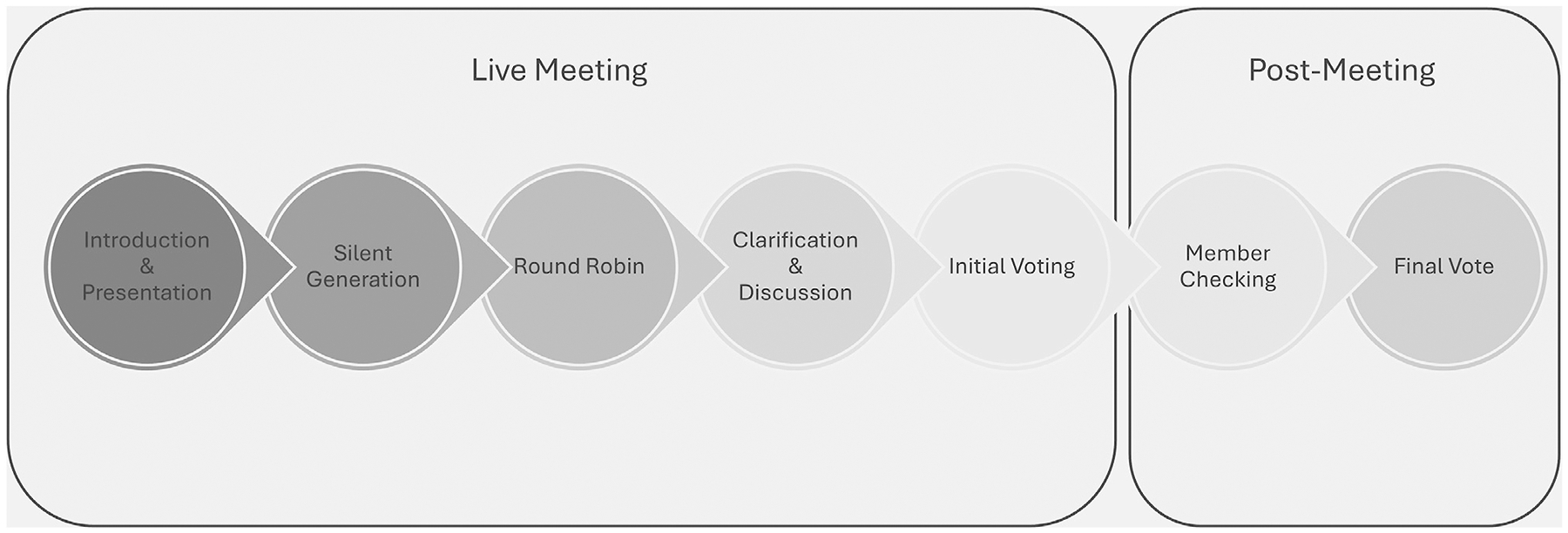
Nominal group consensus technique process. This nominal group consensus study consisted of two phases—live meeting and post meeting. During the live meeting, there were five steps. First, during introduction and presentation, we presented the meeting goals and original Nurse Practitioner Integration Model. In silent generation, the participants had 5 minutes to review the model and write down their thoughts. During round robin, the participants each voiced their key points. In clarification and discussion, the participants had open debate and live revisions to the model. Finally, the participants each voted on initial consensus. The post meeting consisted of member checking in which the participants received the revised model and provided feedback. A final consensus vote was held for the final, revised model.

This qualitative, descriptive expert consensus study used a nominal group consensus technique (NGCT).^19^ NGCT is a highly structured group interaction that facilitates expert discussion and debate.^19,22^ NGCT can be used for several purposes, such as to set research priorities or refine existing thoughts/ideas.^22,23^ We selected NGCT for the latter reason—to refine an existing model. A strength of the NGCT is balanced participation from group members—the session is structured to ensure that each member vocalizes thoughts and votes on agreeability with the output. The NGCT prescribes a single meeting. The typical NGCT consists of four stages—1) silent generation, 2) round-robin idea sharing, 3) clarification and discussion, and 4) voting.^24^ We added two additional stages—member checking and final voting to enhance the rigor of the results and allow participants time to review and respond to the final iteration of the model (Fig. 1).

### Participants & recruitment

In expert consensus studies, participants are chosen based on convenience or purposive sampling.^26^ Experts were considered scholars specializing in integrating NPs into older adult care teams in the United States. To be included in the study, the scholar must have had at least five peer-reviewed publications in the topic area.^20^

NGCT studies usually require 4–14 participants.^19,27^ An advisory panel nominated 62 experts who met the inclusion criteria.^20^ The study team reviewed nominations to ensure that nominees met inclusion criteria; 41 were deemed eligible based on inclusion criteria. The study team sent an e-mail invitation explaining ethical considerations, time commitments, and compensation. Two follow-up attempts were made two weeks apart. We then sent the agreeable participants a scheduling inquiry. Our final sample size included seven participants who agreed to participate and could convene for the NGCT meeting at the same time.

### Data collection

The NGCT meeting was held via video conferencing on because participants were in different geographic locations across the United States. The NGCT meeting occurred on May 10, 2024, which was the only time within the month at which all seven participants were able to attend. The meeting lasted for 2.5 hours, which is within the recommended 2–4 hours.^19,28^

Fig. 1 shows the NGCT process. The meeting began with a presentation of the NP Integration Model and meeting objectives. While delineating objectives, we asked the participants to focus specifically on NP integration for older adult primary care populations. Next, the participants spent 20 min conducting silent generation—i.e., reading, reviewing, and making notes on the NP Integration Model. Participants were then given 5 min to express their ideas during the round-robin session. The next part was open clarification and discussion. The facilitator led the participants through each part of the model under discussion—antecedents, attributes, consequences, illustrative model, and definition. The participants suggested changes. One research team member took notes, and another made real-time changes and updates to the model components, which the participants viewed via “screen share.” In the end, a consensus vote was held to assess whether consensus was reached regarding the changes to the model. Consensus was set at 70 %.^20^

After the meeting, the model was sent to each participant via e-mail for member checking on May 23, 2024.^29^ Minor changes were made, and the participants again received a final voting e-mail on June 6, 2024, which asked whether they agreed with the final model iteration. There was quick turnaround between the NGCT meeting and follow-up inquiries to: 1) ensure that the NGCT meeting content remained recent in the participants’ memories; and 2) increase the likelihood of follow up.^20^ Accordingly, all participants responded during these follow-up stages.

### Data analysis

Data analysis occurred, for the most part, during the NGCT meeting.^19^ In a sense, the participants provided and analyzed the data together. The meeting was recorded and transcribed. The research team reviewed the meeting transcript to ensure that all changes discussed were made.^22^ After sending the revised model for member checking, participants provided additional feedback.^22^ Two team members reviewed the feedback independently and made suggested revisions.^29^ The two team members met to review their interpretation of the changes. There was one disagreement, which was resolved with the rest of the research team.

### Trustworthiness

To enhance the study’s trustworthiness, we followed the criteria established by Guba.^30^ Reflexivity was maintained by documenting personal, interpersonal, methodological, and contextual thoughts and potential biases.^31^ Credibility was enhanced through participant and investigator triangulation.^32^ For dependability, we maintained an audit trail of all changes, which provided a clear record of the study’s evolution and decisions.^33^

### Ethical considerations

The Institutional Review Board of the University of Pittsburgh deemed the study as not human subject research. Nevertheless, steps were taken to protect participants’ identities. Data were stored on a protected, university-based server.

## Results

### Participant characteristics

Table 1 shows participant characteristics. Four of the seven participants had 10 or more years of experience as experts in NP integration. Most participants were 30 to 49. Five of the seven participants worked in a university setting.

### Revised NP Integration Model components

#### Model illustration

**Fig. 2.**
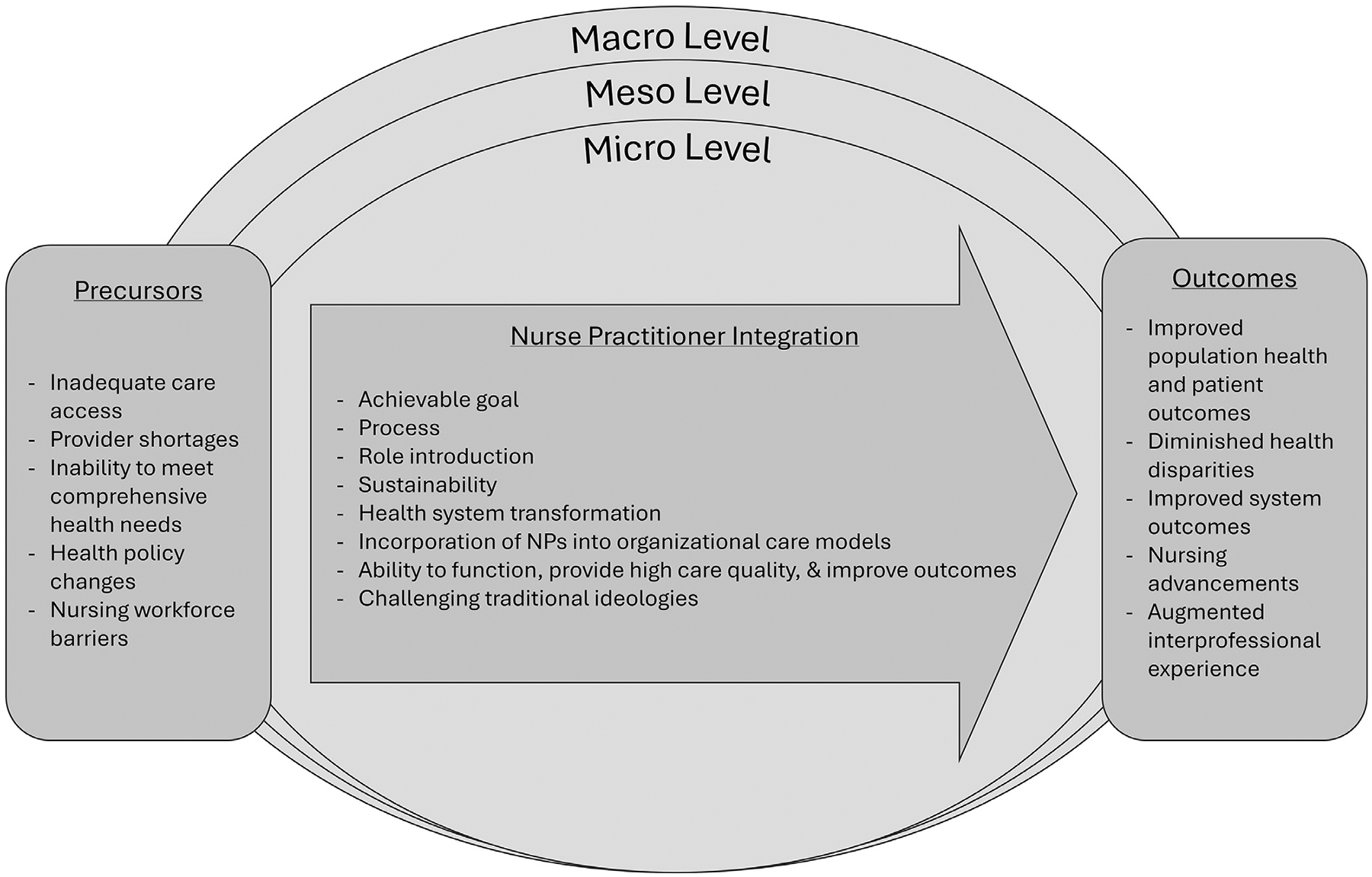
Nurse practitioner integration model. The Nurse Practitioner Integration Model reflects the relationship between the precursors, integration process, and outcomes. The precursors reflect the reasons for incorporating nurse practitioners into older adult primary care teams. Once introduced, the integration process begins. Facilitators and barriers impact integration progress.^25^ At the end of the process, the desired outcomes are achieved. The nurse practitioner integration process, precursors, and outcomes span three health system levels—*macro, meso*, and *micro. Macro* is national/jurisdictional, *meso* is organization (health system or health management organization), and *micro* is the care team level.

Fig. 2 displays the revised NP Integration Model illustration. The precursors reflect the typical impetuses for incorporating NPs into care teams. Next, the process of integrating NPs into the older adult primary care model begins. Facilitators and barriers impact the progress of the NP integration process. At the end of the integration process, the desired outcomes for older adults and the workforce are attainable. The NP integration process, the precursors, and the outcomes span three health system levels—*macro, meso*, and *micro. Macro* represents the national or jurisdictional level. *Meso* represents the organizational level, such as a hospital system or health system. The *micro* level represents the individual care team, typically at the practice level.

#### Definition

All participants agreed on a revised definition of NP integration: a multi-level process of incorporating NPs into a care model to the extent that they can function to their full scope of practice and education, which leads to improved patient, health system, and population needs.

#### Precursors

The precursors represent the factors preceding the integration of NPs into older adult primary care models. Six of the seven participants (86 %) agreed with the list and their descriptions. The five precursors are inadequate care access, provider shortages, inability to meet comprehensive health needs, health policy changes, and nursing workforce issues. Table 2 contains the descriptions of each precursor.

#### Outcomes

Outcomes are the expected results of integrating NPs into older adult primary care teams. All seven participants reached a consensus on the outcomes and their descriptions. The five outcomes are improved population health and patient outcomes, diminished health disparities, improved system outcomes, nursing professional advancement, and augmented interprofessional experience. Table 3 describes the five outcomes.

#### Attributes

The goal of the attributes is to describe the key facets of the concept of NP integration in the older adult primary care context. A unanimous consensus was reached on the eight attributes of NP integration in older adult primary care settings and corresponding descriptions (Table 4). The eight attributes are 1) achievable goal, 2) process, 3) role introduction, 4) sustainability, 5) health system transformation, 6) incorporation of NPs into organizational care models, 7) ability to function, provide high-care quality and improve outcomes, and 8) challenging traditional ideologies.

## Discussion

In this study, experts agreed on a refined NP Integration Model for older adult primary care settings. The model includes precursors, outcomes, attributes, a definition, and an illustrative model of NP Integration into older adult primary care settings. These five components span three health system levels: national/jurisdictional (*macro*), organizational (*meso*), and care team (*micro*).

There were several key changes between the initial NP Integration Model^14^ and this model tailored to older adult primary care. First, the participants recommended tailoring much of the language in the precursor and outcome descriptions to older adults, such as including “across the care continuum.” Second, the illustrative model was revised to enhance user-friendliness. Third, while many existing components of the model, such as the attributes, remained the same after this study, they were still reviewed by all study participants and found to be applicable to older adult primary care. This result is not unexpected as the attributes in the original NP Integration Model diction were phrased for broad applicability across settings and populations^14^

### Older adult primary care population and Nurse Practitioner Integration Model

Today’s healthcare ecosystem challenges older adult primary care NPs to deliver more complex primary care, to more patients, and with more strained resources.^34^ To deliver high-quality primary care for older adults, NPs are expected to incorporate a complex interplay of medical, psychological, social, and environmental factors, which all affect older adult well-being.^35^ Moreover, as primary care standards continue to emphasize patient-centered, holistic care, the number of older adult patients requiring complex primary care is increasing, and the amount of money expended on primary care by government payor programs (e.g., Medicare, Medicaid, Veterans Affairs Health Care, and Indian Health Services) has stagnated.^36^ The National Institute on Aging has recognized this policy conundrum and recommended developing workforce-level interventions to ensure optimal and efficient care delivery for older adults^37^

Herein lays the key purpose of the NP Integration Model tailored to older adult primary care—to improve the efficaciousness of NP care in older adult primary care settings. For one, the model can guide managers and clinicians in integrating NPs into older adult primary care settings. Beyond being an informative tool, the NP Integration Model can be used to guide future research. As it stands, it is known that there are several barriers to NP integration, though there has been little research measuring the impact of these barriers.^8,14^ Research, moreover, has yet to develop interventions addressing existing barriers to NP integration. The model can be used to conceptualize such research studies on NP integration in older adult primary care settings (Table 5). After these studies are conducted, the NP Integration Model could be revised again to incoporate: 1) policy intervention options mapped to existing barriers to NP integration, and 2) a tool to measure NP integration.

### Conceptualizing nurse practitioner integration as a mediator

The NP Integration Model contrasts most published research on NP outcomes in primary care settings by suggesting that an NP integration variable should be included when assessing the relationship between the presence of NPs in a primary care practice and older adult patient outcomes.^4,38,39^ Some studies have measured aspects included in NP integration, such as the work environment and state-level scope of practice.^38,40^ Though, no work has treated these variables as mediators between the presence of NPs in primary care practices and older adult outcomes. Further, the concept of NP integration encompasses more constructs and health system domains (i.e., *macro, meso*, and *micro* levels) than the work environment, scope of practice, and national education policies alone. If empirical research confirms our supposition that NP integration is a mediator, researchers may need to include a measure/variable of NP integration when investigating NP outcomes.

If the data show that NP integration is a mediator, there would be several *macro*- and *meso*-level policy implications to enhance the impact of NP care for older adults in primary care settings.^41^ On the *macro* level, there would be implications for scope of practice, insurance reimbursement structures, transition to practice programs.^40,42^ At the *meso* level, there would be implications for organizational planning and policy evaluation.^43^ *Micro*-level implications focus on interprofessional relationships and work environment issues.^44^

Perhaps NP integration has not been studied as a mediator variable because researchers have yet to validate such a measure encompassing *macro*-, *meso-*, and *micro*-level factors. Empirical research has demonstrated preliminary indicators of internal validity of an NP integration measure encompassing all three health system levels.^25^ The measure, however, still requires psychometric, reliability and validity testing.^25^

### Nurse practitioner integration and implementation science

The NP Integration Model mirrors many concepts in implementation science theories, models, and frameworks.^25^ Implementation science focuses on the implementation of evidence-based interventions into practice in a systematic manner.^45^ We posit that this model frames NP integration as an implementation science topic—evidence shows that introducing NPs into primary care models effectively improves outcomes.^11^ To achieve the evidence-based outcomes associated with the presence of older adult primary care NPs, policies must support the implementation of NPs in an efficacious manner.^41^ Integration is used in the model, instead of implementation because: 1) the literature refers to the process as integration; and 2) integration highlights the complete incorporation of a key workforce member.^11^

Another aspect aligned with implementation science is the multi-level nature of NP integration—i.e., *macro, meso, and micro*. The Consolidated Framework for Implementation Research similarly discusses the role of external and internal factors when implementing an evidence-based intervention.^18,46^ Multi-levelness indicates complexity and whom to target when developing policy to advance NP integration in older adult primary care settings.^41^ In health services research, models are usually meant to inform policy, which echoes a practical benefit of the NP Integration Model.^47–49^

### Implications for research and policy

The refined NP Integration Model targeted at older adult primary care settings can be used for empirical investigation and further theory development (Table 5). Early theoretical development, such as that reported in this study, facilitates describing phenomena and their relationships to other phenomena.^50^ Understanding the relationships between phenomena plays a key role in forming research questions.^50^ Results from empirical investigation inform revised theory.^50^ Accordingly, further theoretical development of NP integration requires empirical investigation.

The NP Integration Model can be applicable for organizational managers to understand NP integration and develop policies to advance integration in older adult primary care settings.^14^ The precursors can be thought of as reasons to integrate NPs. The attributes are aspects with which managers can enhance their prior knowledge of the facets of integration, such as the idea that it is a *process*. The outcomes are the desired end goals, such as improved patient outcomes. These components of the model can help policy decision-makers better understand NP integration.

Finally, consistent definitions must be used in research so that researchers conducting systematic literature reviews can synthesize high-level evidence for consumption by policymakers and organizational decision-makers.^50^ The concept of NP integration, however, is used inconsistently across the literature.^14^ The definition of NP integration resulting from this study provides a definition for researchers to use consistently across studies.

### Strengths and limitations

The study had several strengths. First, NGCTs facilitate data gathering from all participants and not only a few because of the round-robin stage.^19^ We also included an additional round of member checking, which enhanced rigor by allowing participants to re-review the final model and provide feedback.^29^ Finally, the study began with an established model, which helped to direct conversation.^20^

NGCT studies face several practical limitations. A primary critique is that the sampling is non-random, which raises questions about the generalizability of the results. However, it has been argued that the goal of NGCTs, being qualitative, is not to achieve generalizability but rather transferability.^51^ Transferability involves providing a detailed description of the phenomenon so that others can evaluate the extent to which the conclusions apply to other times, contexts, and populations.^29^ Additionally, participant composition significantly influences NGCT studies. Participants are often selected based on convenience and specific criteria, rendering the sample non-random.^20^ While this approach can introduce bias, ensuring a heterogeneous sample can enhance transferability by incorporating diverse perspectives. We strived for heterogeneity in our sample to mitigate biased responses.^28^

## Conclusions

The refined NP Integration Model describes integrating NPs into older adult care primary care settings. The model shows that NP integration may act as a mediator between introducing NPs into a care model and the desired outcomes of their care. The model can be used to guide research testing this hypothesis through empirical research.

## Figures and Tables

**Table 1:** Participant characteristics.

Characteristic	No., %
Total	7 (100%)
Years of Experience	
0–10	2 (29%)
11+	5 (71%)
Age	
30–49	4 (57%)
50+	3 (43%)
Work Setting	
University	5 (71%)
Healthcare Setting	2 (29%)

This table shows the characteristics of experts who participated in the nominal group consensus meeting.

**Table 2:** Precursors.

Precursors	Description
Inadequate care access	Patients often experience gaps in coverage or long wait times to access care. This includes the time it takes to schedule a face-to-face appointment and the wait time at the care facility. Inadequate access to care is common, particularly in primary care clinics and emergency departments.
Provider shortages	There is a shortage of care providers and an unequal distribution of them to meet the population’s care needs. Both the provider workforce and the population are aging, which can drive shortages among the entire workforce or specific specialties.
Inability to meet comprehensive health needs	The supply of care services and expertise lag the increased demand for patient and population health needs across the care continuum.
Health policy changes	National policy changes impact care organizations’ ability to meet care demands at the local level. Policy changes can include changes in work-hour requirements, efforts to improve patient outcomes (e.g., decreasing length of stay and readmissions), and changes in public and/or private health insurance coverage.
Nursing workforce issues	Efforts to improve nursing job outcomes include greater autonomy, a personalized role identity, reduced burnout, enhanced well-being, and better application of knowledge and expertise in practice.

Precursors represent the factors preceding the integration of nurse practitioners into older adult primary care teams. Any one or more may be a reason to introduce nurse practitioners into the care team.

**TABLE 3:** Outcomes.

Outcomes	Description
Improved population health and patient outcomes	Indicators of high value and cost-effective patient and population outcomes associated with NP care. Examples include decreased length of stay, decreased complication rates, decreased rates of morbidity, decreased risk of mortality, improved care continuity, improved patient safety, more patient contact hours, and higher patient satisfaction.
Diminished health disparities	Increased ability for a health system to supply high-quality care options tailored to underserved and under-represented patients and populations.
Improved system outcomes	Indicators of improved health system efficiency and quality (e.g., reduced costs, shorter wait times for an appointment, provider shortage alleviation, improved care access, and decreased unnecessary hospital and emergency department use).
Nursing professional advancement	Development of the nursing workforce to advance education opportunities, advocate for policy changes, and enhance the visibility of nurses in the healthcare team and society.
Augmented interprofessional experience	Indicators of improved interprofessional teamwork and collaboration for all members of the healthcare team (e.g., improved job satisfaction, decreased provider workload, increased provider retention, improved resource utilization, improved clinician well-being, and improved evidence-based care delivery).

Outcomes are the expected results of introducing and integrating nurse practitioners into care teams.

**TABLE 4:** Attributes of Nurse Practitioner Integration.

Attribute	Description
Achievable goal	Nurse practitioner integration was described as having an end goal of full or complete incorporation into care models. Once nearing the achievement of full nurse practitioner integration, nurse practitioners can begin to improve care quality and access significantly for the population which they service.
Process	Nurse practitioner integration occurs over a span of time as opposed to an instance. The process was described as iterative and requiring change, inspiration, acceptance, and intervention. Throughout the process, there is change, role development, policy revision, acceptance, and overcoming challenges. The length of the nurse practitioner integration process is affected by certain factors (i.e., facilitators and barriers).
Role introduction	Nurse practitioner integration is initiated by the introduction of the nurse practitioner role. Effective role introduction often involves organizational and/or government communication of the nurse practitioner role so that the healthcare workforce and patients are aware of nurse practitioner presence and abilities distinct from physicians, physician assistants, and non-prescribing nurses. Moreover, role introduction involves policymaking to expand the care provider workforce by establishing the nurse practitioner role.
Sustainability	The integration of nurse practitioners requires a change in the care model structure to accommodate and incorporate nurse practitioners (i.e., legislative changes permitting licensed nurse practitioners to provide and prescribe care). In more developed care models, legislative and/or regulatory action from government healthcare authorities are often required to guide organizations, nurse practitioners, and healthcare professionals on nurse practitioner scope of practice.
Health system transformation	Nurse practitioner integration may lead to health system transformation. Where integration is more advanced, nurse practitioners are utilized increasingly in primary care with physicians more focused on acute care. Nurse practitioners, thus, may cause a shift in care specialization and health system structures.
Incorporation of nurse practitioners into organizational care models	Nurse practitioner integration involves the addition and inclusion of nurse practitioners in healthcare teams to a point at which nurse practitioners, providers, and other team members view nurse practitioners as care partners. When successfully incorporated, nurse practitioners can function autonomously within the bounds of their scope. When nurse practitioners are incorporated into care models, it becomes clear to the care team that nurse practitioner practice is grounded in nursing practice, which creates a unique niche within care teams.
Ability to function, provide high care quality, & improve outcomes	This attribute is the capacity for nurse practitioners to improve patient outcomes and care quality. Nurse practitioners cannot maximally contribute to care without incorporation into organizational care models. Moreover, this attribute is linked to *achievable goal* as much of the purpose for nurse practitioner integration is to improve care and system outcomes.
Challenging traditional ideologies	Nurse practitioner integration involves interprofessional, intra-professional, and societal rethinking of nursing roles to accommodate expansion of the practice scope. Within care model hierarchies, nurses are often viewed as non-prescribing clinicians who, in addition to nursing care, carry out physician orders. Nurse practitioners introduce alterations to this conventional ideology. The public and healthcare professionals must adjust their view of nurses to accommodate an expanded scope.

Attributes are inherent aspects of nurse practitioner integration.

**TABLE 5:** Using the Nurse Practitioner Integration Model in older adult primary care settings.

Research Question	How to Apply the Model
Are nurse practitioners indicated in my care setting?	Using the precursors (Table 2), it can be determined if the common impetuses for introducing nurse practitioners in a care setting exist.
To what extent did changing integration policies help improve nurse practitioner-linked outcomes in my care setting?	Pre-posts tests ofthe model’s outcomes (Table 3) can be assessed before and after changing policies that impact the integration of nurse practitioners in a care setting.
Have we met the desired outcomes of integrating nurse practitioners in my care setting?	The presence, absence, or extent of outcomes (Table 3) from this model could be assessed.
To what extent do key actors in my care setting understand integrating nurse practitioners?	A study assessing the extent of knowledge on the model’s attributes (Table 4) could be undertaken.

Each potential research question is linked to a component of the model to assist in its practical application.

## Data Availability

The data that support the findings of this study are available on request from the corresponding author. The data are not publicly available due to privacy or ethical restrictions.

## Abbreviations

NP: Nurse practitioner
